# S5-3 Towards national monitoring of physical literacy in Denmark

**DOI:** 10.1093/eurpub/ckad133.025

**Published:** 2023-09-11

**Authors:** Peter Elsborg, Paulina S Melby, Mette Lindholm Kurtzhals, Knud Ryom, Glen Nielsen, Peter Bentsen

**Affiliations:** Copenhagen University Hospital – Bispebjerg and Frederiksberg, Center for Clinical Research and Prevention, Denmark; Copenhagen University Hospital – Bispebjerg and Frederiksberg, Center for Clinical Research and Prevention, Denmark; Copenhagen University Hospital – Bispebjerg and Frederiksberg, Center for Clinical Research and Prevention, Denmark; Department of Public Health, Aarhus University, Denmark; Department of Nutrition, Exercise and Sports, University of Copenhagen, Denmark; Copenhagen University Hospital – Bispebjerg and Frederiksberg, Center for Clinical Research and Prevention, Denmark

## Abstract

**Purpose & Method:**

This presentation will focus on the Danish context and will present how physical literacy (PL) is culturally different in Denmark, illustrated by describing how a translation and context adaptation process of the Canadian Assessment of Physical Literacy (CAPL-2) led to the development of ‘MyPL’ – a video-assisted questionnaire that assesses Danish children’s PL self-reported. Primary focus will be on how this questionnaire has been the first step towards national monitoring of the Danish population’s PL.

**Results:**

During the developmental process of MyPL, researchers from two different Danish universities initiated a collaboration with the Danish Institute of Sport Analysis, resulting in the inclusion of a version of MyPL in their representative survey covering the Danish population’s sport and exercise habits and thus providing a unique opportunity to monitor the population’s PL levels over time.

**Conclusions:**

The results from this survey will contribute to our understanding of PL in Denmark and further provide valuable insights into how PL levels in the Danish population, most efficiently, is monitored. This presentation will argue for the potentials of a national monitoring of PL and provide valuable insights into the Danish context and the status of PL amongst the Danish population.

